# Spindles in Svarog: framework and software for parametrization of EEG transients

**DOI:** 10.3389/fnhum.2015.00258

**Published:** 2015-05-08

**Authors:** Piotr J. Durka, Urszula Malinowska, Magdalena Zieleniewska, Christian O'Reilly, Piotr T. Różański, Jarosław Żygierewicz

**Affiliations:** ^1^Faculty of Physics, University of WarsawWarsaw, Poland; ^2^Department of Neurology, Epilepsy Center, Johns Hopkins University School of MedicineBaltimore, MD, USA; ^3^McConnell Brain Imaging Center, Montreal Neurological Institute, McGill UniversityMontreal, QC, Canada; ^4^Center for Advanced Research on Sleep Medicine, Centre de Recherche de l'Hôpital du Sacré-Cœur, Université de MontréalMontreal, QC, Canada; ^5^College of Inter-Faculty Individual Studies in Mathematics and Natural Sciences, University of WarsawWarsaw, Poland

**Keywords:** sleep spindles, matching pursuit, EEG transients, time-frequency, sleep, Svarog, open source, free software

## Abstract

We present a complete framework for time-frequency parametrization of EEG transients, based upon matching pursuit (MP) decomposition, applied to the detection of sleep spindles. Ranges of spindles duration (>0.5 s) and frequency (11–16 Hz) are taken directly from their standard definitions. Minimal amplitude is computed from the distribution of the root mean square (RMS) amplitude of the signal within the frequency band of sleep spindles. Detection algorithm depends on the choice of just one free parameter, which is a percentile of this distribution. Performance of detection is assessed on the first cohort/second subset of the Montreal Archive of Sleep Studies (MASS-C1/SS2). Cross-validation performed on the 19 available overnight recordings returned the optimal percentile of the RMS distribution close to 97 in most cases, and the following overall performance measures: sensitivity 0.63 ± 0.06, positive predictive value 0.47 ± 0.08, and Matthews coefficient of correlation 0.51 ± 0.04. These concordances are similar to the results achieved on this database by other automatic methods. Proposed detailed parametrization of sleep spindles within a universal framework, encompassing also other EEG transients, opens new possibilities of high resolution investigation of their relations and detailed characteristics. MP decomposition, selection of relevant structures, and simple creation of EEG profiles used previously for assessment of brain activity of patients in disorders of consciousness are implemented in a freely available software package Svarog (Signal Viewer, Analyzer and Recorder On GPL) with user-friendly, mouse-driven interface for review and analysis of EEG. Svarog can be downloaded from http://braintech.pl/svarog.

## 1. Introduction

Sleep spindles are defined in Rechtschaffen and Kales (1968); Ibert et al. (2007) as a train of distinct waves with frequency 11–16 Hz (most commonly 12–14 Hz) with a duration ≥ 0.5 s Detection of these structures by human experts, trained in visual analysis of EEG, constitutes a gold standard. Unfortunately, the inter-expert agreement in scoring sleep spindles is limited. This drawback undermines the idea of repeatability of experiments, which lies at the foundations of hard sciences: the same study of sleep spindles on the same dataset may yield different results, because of differences in the visual selections done by human experts.

Explosion of the applications of computerized signal processing methods resulted in a multitude of automatic detection algorithms. The most effective so far are based upon a common framework, introduced in Schimicek et al. (1994), reviewed e.g., in Warby et al. (2014):

1. EEG is band-pass filtered in the frequency range related to sleep spindles.
2. Signal from the previous step is subjected to amplitude thresholding in the time domain.
3. Epochs exceeding the threshold are filtered in the time domain to select those corresponding to sleep spindles.

Contrary to the visual detection by human experts, who concentrate directly and separately on relevant transient structures visible in EEG, each step of this sequential procedure implements only one aspect of the definition, and accumulates the bias from the previous steps. This drawback is the consequence of separate application of filters in the frequency and time domains. This turns our attention to the time-frequency methods of signal processing.

Classically, methods like short-time Fourier transform (STFT) and wavelet transform (WT) are used to compute the distribution of signal's energy in the time-frequency plane (Durka and Blinowska, 1997). Regions of increased energy correspond directly to signals transients, but their automatic selection still requires some kind of thresholding. Bias resulting from *a priori* choices of thresholds and further postprocessing becomes even more difficult to assess than in the spectral methods. Also, results depend significantly on prior choices of parameters like the duration of the time window in STFT or choice of the mother wavelet in WT.

**Figure 1:**
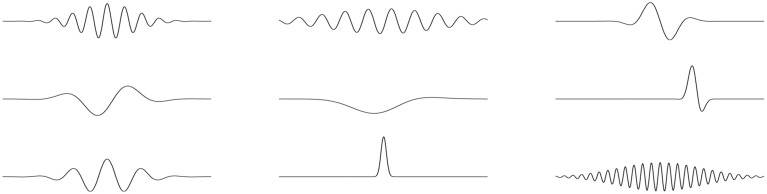
**Examples of Gabor functions, defined as Gaussian envelopes modulated by sinusoidal oscillations**.

Algorithm adapting the parameters automatically to the local content of the analyzed signal was introduced in Mallat and Zhang (1993). Matching pursuit algorithm (MP, Section 2.1) is an iterative procedure explaining the signal as a sum of Gabor functions (Figure 1), chosen optimally from a large and redundant set. Comparing to WT and STFT, analysis window and partly also the mother wavelet in this approach are chosen individually for each local transient structure present in the analyzed signal. Another unique feature of MP is the explicit parameterization of the structures fitted to the signal in terms of their time and frequency centers, duration and phase. This allows to perform detection directly in the space of these parameters in one step.

This approach has been successfully applied for the detection and parameterization of EEG transients including sleep spindles in different paradigms, mostly at the University of Warsaw. Additionally, MP-based detection of several types of EEG transients can be efficiently combined into an automatic sleep stager, based explicitly upon the accepted criteria for stages (Malinowska et al., 2009). However, in spite of almost 20 years of publishing results (c.f. Durka and Blinowska, 1995; Żierewicz et al., 1999; Malinowska et al., 2013 and many more) and free software for MP decomposition (our versions of the MP algorithm have been freely available since 2001, Durka et al., 2001), this approach to EEG analysis has been seldom applied outside our group. One of the reasons may have been a relative technical complexity of the whole procedure. To cope with this problem, this paper introduces a user-friendly and freely available multiplatform software for detection of sleep spindles (and other transients) in MP decompositions of EEG. This plugin is embedded in Svarog—Signal Viewer, Analyzer and Recorder On GPL.

Detection of sleep spindles presented in this paper relies on the correspondence of their shape (waxing and waning oscillations) to the Gabor functions used in MP decomposition (Figure 1), so finding corresponding structures among the Gabor functions fitted by the MP to EEG time series is straightforward and consists of setting the limits on their frequency centers, durations and amplitudes. Duration and frequency are taken literally from the definition of sleep spindles. As for the minimal amplitude, which is not directly defined, we adapt the common approach, which relates this parameter to the RMS of the signal filtered in the sigma band.

## 2. Materials and methods

### 2.1. Matching pursuit algorithm (MP)

#### 2.1.1. Matching pursuit (MP)

MP was proposed by Mallat and Zhang (1993) as a suboptimal, iterative solution to the intractable problem of an optimal representation of a signal *x* in a redundant dictionary *D*, containing dense set of functions *g*_γ_. In plain English, the gist of the MP procedure can be summarized as follows:

1. We start by creating a huge, redundant set *D* (called a dictionary) of candidate waveforms for representation of structures possibly occurring in the signal. For the time-frequency analysis of signals we use dictionaries composed of sines with Gaussian envelopes, called Gabor functions, which reasonably represent waxing and waning of spindle oscillations.
2. From this *D* dictionary we choose only those functions, which fit the local signal structures. In such a way, the width of the analysis window is adjusted to the local properties of the signal. Local adaptivity of the procedure is somehow similar to the process of visual analysis, where an expert tends to separate local structures and assess their characteristics. Owing to this local adaptivity, MP is the only signal processing method returning explicit time span of detected structures.
3. The above idea is implemented in an iterative procedure: in each step we find the “best” function, and then subtract it from the signal being decomposed in the following steps.

As for the mathematical description, denoting the function fitted to the signal *x* in the *n*-th iteration of MP as *g*_γ_*n*__, and the residual left after *n*-th iteration as *R*^*n*^*x*, we can describe the procedure as:

(1) $$\{\begin{array}{l} R^{0}x=x\\ R^{n}x=\langle R^{n}x,g_{\gamma_{n}}\rangle g_{\gamma_{n}}+R^{n+1}x\\ g_{\gamma_{n}}=\arg\max_{g_{\gamma_{i}}\in D}|\langle R^{n}x,g_{\gamma_{i}}\rangle | \end{array}$$

where 〈·, ·〉 denotes the inner product of signals and | · | the absolute value. As a result we get an approximate expansion:

(2) $$x\approx \sum_{n=0}^{M-1}\langle R^{n}x,\;g_{\gamma_{n}}\rangle g_{\gamma_{n}}$$

where *M* equals the number of iterations of Equation (1). For a time-frequency analysis of real-valued signals, dictionary *D* is usually composed from Gabor functions:

(3) $$g_{\gamma }(t)=K(\gamma )e^{-\pi {\left(\frac{t-u}{s}\right)}^{2}}\cos\left(\omega (t-u)+\phi \right)$$

where γ is a set of parameters such that γ = (*u*, ω, *s*) and *K*(γ) is a normalization constant such that ||*g*_γ_|| = 1.

The procedure is generic. The only major settings correspond to:

- quality of the decomposition, regulated mainly by the size of the dictionary *D*, which controls the parameterization accuracy of detected structures.
- number of iterations *M*, which regulates the accuracy of the overall approximation, the number of low energy structures included in the decomposition increases with *M*.

In both cases, higher settings result in higher accuracy.

#### 2.1.2. Size of the dictionary *D*

**Figure 2:**
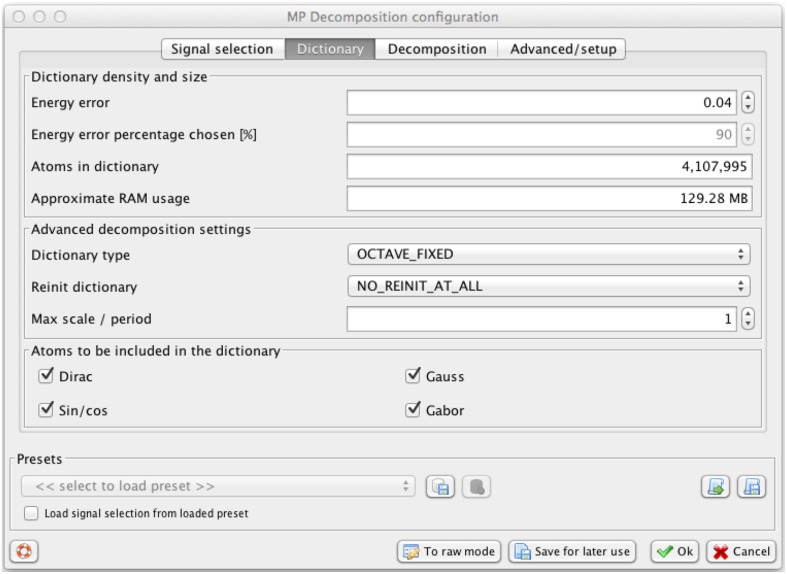
**Svarog window for setting the parameters of the MP decomposition, presenting values used in this study**. Decreasing the parameter “Energy error”—in the text referred as ϵ—increases resolution and the number of functions in the dictionary and usage of RAM, calculated automatically in the lower panels. For explanation of other parameters see Kuś et al. (2013).

Size of the dictionary *D* determines the number of candidate waveforms that will be fitted to the signal, and hence the resolution of the resulting decomposition. The resolution goes up with the number of functions in the dictionary. To make this setting independent of the size of the signal, we introduced one parameter regulating the density of the dictionary, related to the maximum distance between the dictionary's waveforms. This parameter is called in the Svarog interface (Figure 2) “energy error” ϵ, since it relates to the maximum error that MP can make in a single iteration, as explained in details in Kuś et al. (2013)^1^.

This special construction of the dictionary, ensuring a uniform distribution in the space of inner products, imposes non-uniform distribution of dictionary's functions in the space of their time positions, widths and frequencies (Kuś et al., 2013). For example, setting of ϵ = 0.04 used for MP decompositions in this paper gives, for the frequency range of sleep spindles, possible time widths 0.53, 0.8, 1.21, and 1.82 s. That means that a spindle—or even a perfect Gabor function—with a width 1.5 s will be matched by a Gabor function from the dictionary with width either 1.21 or 1.82 s, and the leftover energy will be accounted for in the remaining iterations or will be left as a residual modeling noise if not accounted by the first *M* functions.

#### 2.1.3. Number of iterations *M*

Number of iterations *M* is easier to assess, since the *g*_γ_*n*__ in Equation (2) are ordered by decreasing energy. That means that in two different decompositions differing only in the setting of the number of iterations, say 50 and 100, the first 50 waveforms will be the same (with small exceptions if stochastic decomposition was chosen), and iterations 51–100 will contain only structures of energy smaller than contributed by *g*_γ_50__.

Increasing the number of iterations will not improve the quality of fit of any single waveform, so if we are interested in structures of relatively high energy, as is usually the case when looking for structures which are also visible for human expert, it makes no sense to increase *M* above the number which can be determined heuristically for a given problem and class of signals.

Described above MP decomposition is a purely mathematical procedure. In relation to EEG analysis, bad news are:

1. Computation of the MP decomposition of a signal is relatively time-consuming even on a modern PC.
2. Settings of the energy error and number of iterations may require some consideration in case of limited computational resources, as discussed above in Sections 2.1.2 and 2.1.3.

Good news are:

1. Unlike most of the time-frequency methods of signal processing, setting of parameters is not a tradeoff between different aspects of the quality of decomposition, but a tradeoff between the quality and speed.
2. MP decomposition is generic, and once performed, the same decomposition of given epoch can be used to investigate the presence of different structures (c.f. **Figures 7**, **8**). That's where the weight is switched from mathematics to neuroscience.

#### 2.1.4. Software implementation

**Figure 3:**
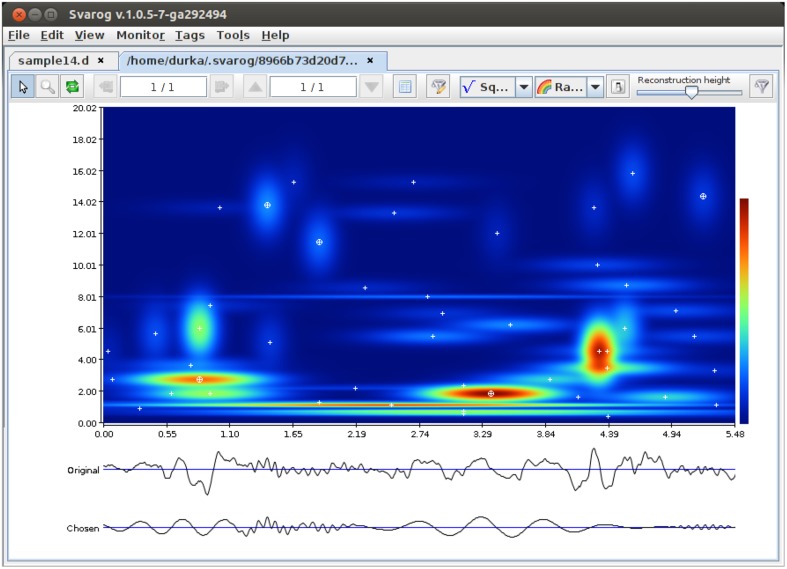
**Results of MP decomposition displayed as an interactive time-frequency map of signal's energy density in Svarog**. Clicking center of a blob (marked by white cross) adds the corresponding function to the reconstruction (bottom signal). From Kuś et al. (2013)

Program computing the actual MP decomposition of given epoch is implemented in C and compiled separately for each platform. It is a command-line program, taking input from a config file and writing output to a binary file containing parameters of the fitted functions (a “book” ^*^.b). To facilitate its application, we created a wrapper/GUI module for Svarog, which is a multiplatform EEG review system. After installation and configuration of the system (Section 4.4), user can perform MP decompositions of the epoch selected by mouse, setting the decomposition parameters in tabs of the window displayed in Figure 2. Svarog then writes the selected (referenced and filtered) epoch to disk and calls the MP binary, which computes its decomposition and saves results to disk. These results can be then explored as an interactive time-frequency map as shown in Figure 3, or used for construction of summary reports on selected structures, as discussed in Section 3.3. For those who want to design their own post-processing, we provide scripts for reading the results of MP decomposition in Matlab and Python (Section 4.4).

### 2.2. Experimental data

Data comes from the first cohort/second subset of the Montreal Archive of Sleep Studies (MASS-C1/SS2) (O'Reilly et al., 2014). It includes whole-night recordings from 19 young and healthy participants (8 male and 11 female; 23.6 ± 3.7 SD years old) with expert scoring of sleep stages according to the rules of Rechtschaffen and Kales (1968). For the gold standard, we used scoring of spindles from expert #1 available on MASS website. This scoring was performed for epochs of non-rapid eye movement stage two sleep, on C3 channel (linked-ear reference), and following AASM rules (Ibert et al., 2007). This database was chosen as it is open for sleep research and therefore facilitate reproducibility (see Section 4.4).

### 2.3. Measures of performance of detection

We based the assessment of efficiency of the detector on the markings with the accuracy of the EEG sampling, as proposed in O'Reilly et al. (in revision). In such an approach, at each sample (in our case 256 samples per second), there are four well-defined outcomes of comparison of expert's and detector's scorings: spindle present according to both expert and detector (true positives; *TP*), spindle absent according to both expert and detector (true negatives; *TN*), spindle present according to expert, but absent according to detector (false negative; *FN*), spindle absent according to expert, but present according to detector (false positives; *FP*). Counts of each type of outcome can be used to formulate various measures of detector performance:

(5) $$\text{sensitivity}=\frac{TP}{TP+FN}$$

Positive predictive value^2^ (PPV):

(6) $$\text{PPV}=\frac{TP}{FP+TP}$$

Matthews coefficient of correlation (MCC):

(7) $$\text{MCC}=\frac{TP*TN-FP\;*\;FN}{\sqrt{P\;*\;P^{\prime }\;*\;N\;*\;N^{\prime }}}$$

where *P* = *TP* + *FN*, *P*′ = *TP* + *FP*, *N* = *FP* + *TN*, *N*′ = *FN* + *TN*.

Cohens κ:

(8) $$\kappa =\frac{\frac{TN\;+\;TP}{P\;+\;N}-P_{e}}{1-P_{e}}$$

where *P*_*e*_ is the probability of random agreement defined as:

(9) $$P_{e}=\frac{P^{\prime }P\;+\;N^{\prime }N}{{\left(P+N\right)}^{2}}$$

F_1_-score:

(10) $${\text{F}}_{1}=2\;*\;\frac{\text{PPV }*\text{sensitivity}}{\text{PPV}+\text{sensitivity}}$$

### 2.4. Detection of sleep spindles

Division between the purely mathematical MP decomposition of signals and further neuroscience research is clearly reflected in the structure of the Svarog software package. The first step, briefly covered in Section 2.1, consists of a generic approximation of the signal by a linear sum of Gabor functions. The second step, which is selection of the structures corresponding to sleep spindles, constitutes the main topic of this article.

MP offers explicit parameterization of signal structures in terms of their time and frequency positions, widths and amplitudes. Detection of sleep spindles within the proposed framework can be perceived as filtering out irrelevant structures from a database containing all the waveforms fitted by MP to a given signal epoch. Settings of the filter can be directly based upon the classical definition(s) mentioned in the Introduction. We choose frequency range 11–16 Hz and duration exceeding 0.5 s. Duration and time center of each detected spindle are returned explicitly by the MP algorithm, as parameters *u* and *s* from Equation (3), which gives us the time extent of the spindle from *u* − *s*/2 to *u* + *s*/2. Duration is taken here explicitly as the half-width of the Gaussian envelope of the Gabor function, but it can be adjusted by a multiplicative factor e.g., to optimize the concordance with visual detection. In general, using the setting window presented in **Figure 7**, one can easily test the procedure with different settings adjusted e.g., to different definitions, like frequency 12–14 Hz as defined in Rechtschaffen and Kales (1968) or slow (11–13 Hz) and fast (13–16 Hz) spindles separately.

**Figure 4:**
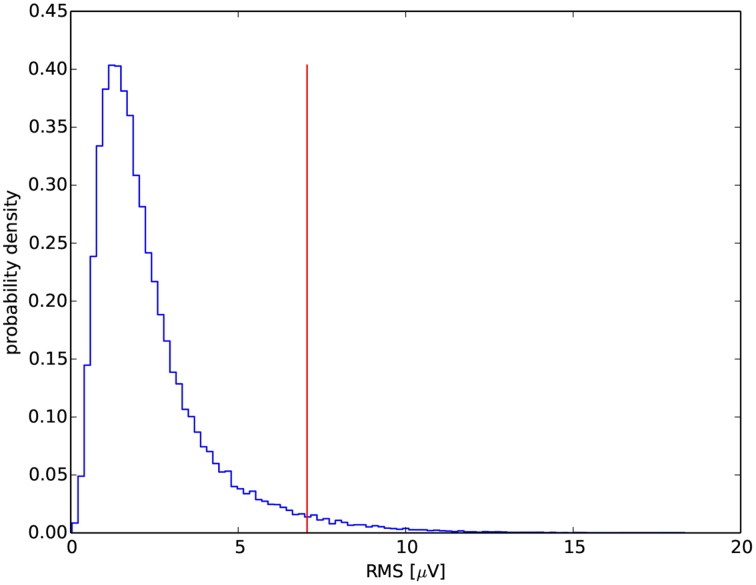
**Exemplary distribution of RMS for one of the recordings**. The vertical line marks its 97th percentile.

Due to the lack of a precise definition of the minimum amplitude for spindles, one can either adapt a fixed threshold (e.g., Schimicek et al., 1994; Ventouras et al., 2005), usually optimized for a given recording (which causes obvious problems with generalization of the procedure to recordings from other labs/cohorts), or compute a threshold based upon the properties of the analyzed signal and in particular adapted to individual subject (e.g., Huupponen et al., 2000; Ray et al., 2010), which results in a more general procedure. We compute this threshold in relation to the RMS distribution. Exemplary distribution for one of the recordings is shown in Figure 4. To obtain the RMS distribution we filter the signal in the frequency band of sleep spindles (using 2nd order band-pass Butterworth filter with the cutoff frequencies set to 11 and 16 Hz). The RMS values were evaluated in successive, non-overlapping time windows with duration of 0.2 s. With this combination of bandwidth and window duration, one window includes more or less one period of oscillations of the filtered signal. Thus, in each window we can assume an approximate relation between amplitude and RMS as for a constant-amplitude sine wave. In such case peak-to-peak amplitude relates to the RMS as:

(11) $$A=2\sqrt{2}P_{\text{RMS}}$$

where *P*_RMS_ is the percentile of the mentioned RMS distribution, chosen to maximize resulting MCC.

## 3. Results

### 3.1. Performance of sleep spindles detection in individual cases

**Figure 5:**
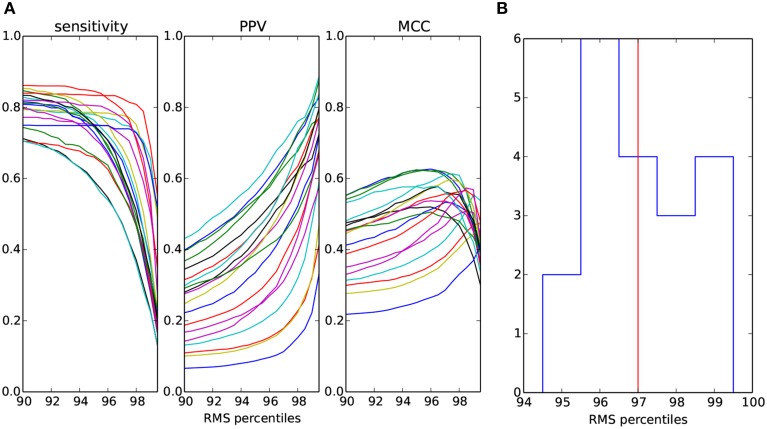
**(A)** Measures of spindles detection quality as a function of the percentile of RMS in the spindle frequency band computed separately for each of the 19 overnight recordings and markd by different colors. **(B)** Distribution (counts) of RMS percentiles which maximize MCC shown in **(A)**. Median of the distribution marked with vertical line.

As described in Section 2.4, the minimal amplitude of candidate waveform is a free parameter in the proposed detector of sleep spindles. In order to have a complete picture of the detector performance on the current dataset, in Figure 5A we present the sensitivity, PPV and MCC for a range of RMS percentiles.

Figure 5B shows the distribution of the optimal, in the sense of maximizing MCC, percentiles for each of the recordings. The median of this distribution is the 97th percentile.

### 3.2. Cross-validation

A common pitfall in the evaluation of the algorithms detecting sleep spindles is their explicit optimization for a particular dataset, often the same as the one used for presenting the performance of resulting algorithm. It is also a common problem in evaluation of detection algorithms, and the standard solution used in machine learning is called cross-validation.

For the evaluation of performance of the proposed method, we implement the following cross-validation procedure, related to the only parameter not taken directly from the definition of sleep spindles, which is the minimal amplitude expressed in terms of the percentile of RMS distribution in the frequency range of sleep spindles:

1. Randomly divide the available recordings in two disjoint subsets, further called the training set and the validation set.
2. Compute the optimal percentile for the training set.
3. Evaluate the performance on the validation set.
4. Repeat steps (1–3).

By averaging resulting performance measures over different random divisions of the available dataset we obtain an estimate of the average performance of the procedure on “unseen” data. This estimate tends to be a bit lower than the overall performance computed and estimated on the whole dataset at once.

**Figure 6:**
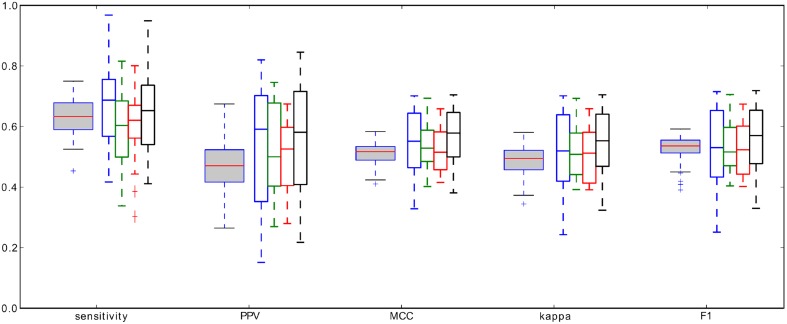
**Gray-filled boxplots: distributions of the average performance measures of spindles detection, defined in Section 2.3, obtained from the cross-validation procedure, white-filled boxplots: performance measures of the four detectors (color coded: red—RMS, green—RSP, blue—Sigma, black—Teager) tested in O'Reilly et al. (in revision) on the same data set**.

We performed 100 iterations of the cross-validation procedure, each time randomly choosing 14 recordings for the training set used to compute the optimal RMS percentile. Then these 14 percentiles *P*_RMS_, optimal for each of the recording separately, were averaged. The resulting average threshold was applied to find the minimal spindle amplitudes for all the remaining 5 recordings. Figure 6 shows the distribution of the resulting performance measures averaged over the validation sets. The summary statistics of performance are presented in Table 1.

**Table 1:** **Summary of cross-validation performance statistics**.

**Measure**	**Median**	**First quartile**	**Third quartile**	**Mean**	**Standard deviation**
Sensitivity	0.63	0.59	0.68	0.63	0.06
PPV	0.47	0.42	0.52	0.47	0.08
MCC	0.52	0.49	0.53	0.51	0.04
Cohen kappa	0.49	0.46	0.52	0.49	0.05
F_1_-score	0.54	0.51	0.56	0.53	0.04

### 3.3. EEG profiles

Proposed approach offers precise detection of time centers and durations of sleep spindles and other transients. Apart from these, MP decomposition provides also an explicit and high resolution parameterization of their frequencies, amplitudes and phases. This opens a simple access to detailed information on the pattern of their occurrences across the whole analyzed recording, including:

- exact time occurrences of each detected structure with information about amplitude of each detected spindle.
- number of structures per epoch (in sleep analysis this is traditionally 20 or 30 s).
- percent of the epoch's time occupied by selected transients.

Although the last parameter has not been used for sleep spindles so far, all these reports are presented for demonstration in the three upper panels of **Figure 8**.

**Figure 7:**
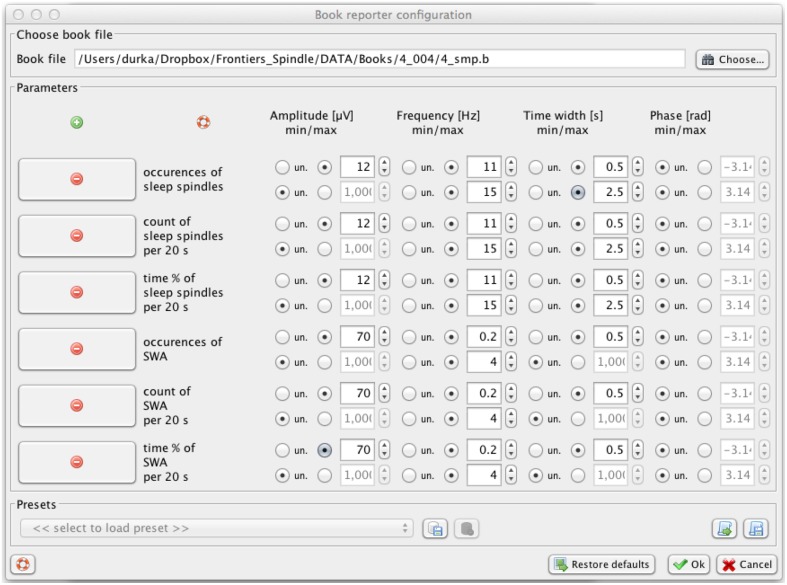
**Svarog window for setting the parameters of filters defining the structures chosen from MP decomposition for the profile presented in Figure 8**. This functionality operates on the results of a previously computed MP decomposition (Figure 2).

Sleep spindles are not the only EEG transients which can be effectively detected and parameterized by means of proposed approach. Another classic example of transient structures crucial for assessment of the sleep process are slow waves (Durka et al., 2005a). Figure 7 presents example parameters allowing for selecting, from the same MP decomposition of the same signal, structures corresponding to slow waves: amplitude above 70 μV, frequency 0.2–4 Hz, and time width above 0.5 s.

**Figure 8:**
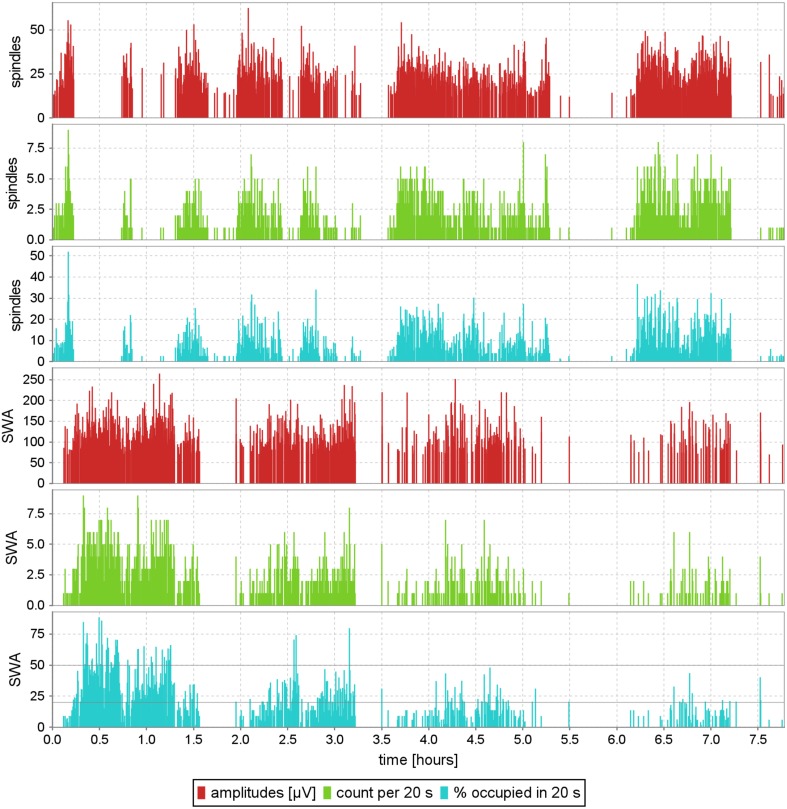
**Exemplary EEG profile of sleep, computed automatically without prior removal of artifacts, for the structures defined in Figure 7; subject #4 of MASS-C1/SS2**. Each of the red vertical lines is positioned at the exact time center of a spindle or slow wave, and its height represents the structure's amplitude. Green lines are positioned in 20-s intervals, and their height measures the number of structures detected in the corresponding epochs. Cyan lines, like the green ones, relate to the properties of 20-s epochs, and give the percent of each epoch's time, occupied by the given structures. This parameter is especially relevant for slow waves, because (Rechtschaffen and Kales, 1968) defined sleep stages 3 and 4 explicitly by 20–50% and 50–100% ranges of this parameter (Malinowska et al., 2009).

Figure 8 presents these profiles for sleep spindles and slow waves, computed in a fully automatic way without prior removal of artifacts. Examples of time-frequency definitions of structures in Svarog also include alpha, beta, theta and delta waves, and K-complexes (Malinowska et al., 2009). As explained in Section 4.1, all these profiles can be computed from the same MP decomposition, and reports for different settings of filters defining these structures, contrary to the underlying MP decomposition, are computed in seconds.

These profiles can be used for investigating several features of EEG, previously assessed by different specially constructed algorithms, or by visual inspection. For example:

- Report in the lower panel of Figure 8, showing the time fraction of each epoch occupied by slow waves, is crucial for sleep staging, since stages 3 and 4 are defined directly in terms of ranges of this parameter (Stage 3: 20–50%, Stage 4: above 50%). This correspondence was explored in Malinowska et al. (2009) for automatic construction of hypnograms, based directly upon the classical criteria from Rechtschaffen and Kales (1968).
- Profiles for these and other structures were used for assessment of the brain activity of patients in different states of disorders of consciousness (Malinowska et al., 2013).

## 4. Discussion

### 4.1. Computational complexity of MP

As mentioned in Section 2.1, in each step of the MP algorithm we compute inner products of all the functions from the dictionary with the signal (or the residuum left from previous iterations). Implemented directly, this would typically result in millions of inner products, each computed on thousands of samples. Such massive computations impose a significant burden even for modern computers. Fortunately, it is possible to decrease it significantly with mathematical and programming tricks. The former, implemented in the current version of the MP algorithm used for computations in this article and available together with Svarog from http://braintech.pl/svarog, are described in Mallat and Zhang (1993) and Kuś et al. (2013). However, this user-friendly software is still a research system, not aimed at commercial applications. Since the speed of computations was not the major goal here, not all the optimizations were explored yet. Also, as discussed in Section 2.1.2, we used a relatively dense dictionary, increasing significantly the computational burden: with 50 iterations per epoch, decomposition of one overnight recording took about 48 CPU-hours. Since the MP5 algorithm is single threaded, we were able to run 11 concurrent instances on a 12-core computer, thus decomposing in average one overnight recording every 4h approximately. While this may still look like a lot of computing time, let us recall that:

1. MP decomposition is performed only once per each analyzed signal, and as such needs not to be interactive. Using one such general decomposition, we can investigate any structures potentially present in the signal (Section 4.3) in a comfortably interactive mode. Results from one channel of an overnight recording like the one presented in Figure 8 are computed in seconds.
2. There is still room for significant speed improvements, in the optimization of code (e.g., multithreading or using GPUs) as well as in the adjustments of the decomposition parameters to a particular problem. As an example of the latter we may quote an online procedure for detection of epileptic seizures in commercial EEG software by Persyst (http://persyst.com, patent US 6735467), based on a previous version of our MP algorithm (Durka et al., 2001).

### 4.2. Performance of detection

Reported performance of sleep spindle detectors depends both on the properties of the detector and on the quality of experts scores. Therefore, the quantitative comparison of detectors is possible only on the same database of EEG recordings and scorings, otherwise the comparison is rather qualitative. It is especially so if the parameters of the detector are tuned to maximize the performance for a given dataset. Another problem in comparison between the results reported in literature is that various authors define the correct detection in different ways via the “window based” type of comparison—mainly in respect to the criteria defining the overlap between detectors and experts scores. We used “signal-sample-based” assessment of performance, since we find it much less ambiguous. In general, the values obtained in “signal-sample-based” type of comparison are more conservative than those obtained in “window based” comparison, as was demonstrated in O'Reilly et al. (in revision). Unfortunately, “window based” comparison is the most common and for a long time was the only one considered for assessing the performance of spindles detection presented in literature. To give a general background we cite below some of the results.

For example, one of the first automated detection method with fixed amplitude threshold (Schimicek et al., 1994) showed sensitivity of 89.7% and a specificity of 93.5%. Other sleep spindles detection method using artificial neural networks (Ventouras et al., 2005) presented the sensitivity of the network ranges from 79.2 to 87.5% and specificity from 88.4 to 97%, with the false detection rate ($FDR=\frac{FP}{FP+TP}$) ranging from 2.1 to 21.5%. The methods where variability of sleep spindles amplitude across subjects have been taken into account for detection (e.g., Bódizs et al., 2009) reported sensitivity of 92.9 and 58.4% false detection rate. Another work (Huupponen et al., 2007)^3^ testing four different detection methods reported optimal sensitivity of 70% for a false detection rate of 32%. Ray et al. (2010) reported a sensitivity of 98.96% for a specificity of 88.49%, with a corresponding 37.2% false detection rate in detection of sleep spindles in stage II with the minimal amplitude adjusted individually and 3 s scoring windows.

A more direct comparison of the detector presented in this work can be made with the six automatic detectors, known from publications, reimplmented and tested in Warby et al. (2014) (cf. Figure 4). The authors presented “precision-recall” plot obtained with “window based” comparison of the detectors^4^. Our detector would be placed at point (recall = 0.63, precision = 0.47) in that space, which is close to the middle of the automated group consensus curve. Also the F_1_-score is close to the maximum performance for the auto group consensus. Such result would indicate that the proposed detector is well balanced and close to optimal among the automated detectors, but we have to keep in mind that we compare results for different datasets.

The most meaningful and direct comparison can be made with the four detectors tested in O'Reilly et al. (in revision), since they were tested on exactly the same data set, with same expert scoring, and using the same “signal-sample-based” type of comparison. For the ease of comparison, in Figure 6, we rearanged the original results presented in O'Reilly et al. (in revision). These detectors were: RMS—based on methodology proposed in Schimicek et al. (1994), RSP—relative spindle power detector based on Devuyst et al. (2011), Sigma—based on the sigma index proposed by Huupponen et al. (2007), and Teager—based on Teager energy operator, as in Ahmed et al. (2009). Comparison of all four classifiers tested by O'Reilly et al. as well as the MP-based classifier presented in this work, shown in Figure 6, have the same range of performance measures, if one takes into account the spread of the distribution of the measures, which in fact is quite broad. In our opinion, this fact points to the limitations of consistency of expert's scorings which were used as the “gold standard,” or to the existence of some characteristics of the recording which affects the decisions of expert, but which are not included in the currently used definition of sleep spindles.

### 4.3. Universal parametrization

In the context of a universal parameterization of EEG transients (Durka, 2005) it is also worth mentioning that proposed framework has a potential to solve a variety of important problems in EEG analysis. Apart from the above examples, it was already shown to significantly improve the quality of EEG inverse solutions if used as a preprocessing and automatic detection of sleep spindles (Durka et al., 2005b), and sensitivity of estimates used in pharmaco EEG (Durka et al., 2002).

We believe that the availability of the free software and exemplary description of a framework for detection of sleep spindles paves the way to novel and creative applications of this high-resolution parametrization, to a large extent compatible with the tradition of visual analysis.

### 4.4. Data sharing

Complete software package (with source code) used in this study for computing MP decompositions and generating Figure 8, as well as scripts for reading the results of MP decomposition in Matlab and Python (Section 2.1.4), are freely available from http://braintech.pl/svarog. Source code of the Svarog interface (in Java) and mp5 program for MP decomposition (in C) is available from http://git.braintech.pl.

Polysomnograms and human scoring of sleep spindles used in this study come from MASS database and can be downloaded from http://ceams-carsm.ca/en/mass. Access to polysomnographic recordings requires further accreditation from an authorized Ethics Research Board.

## Author contributions

PD has proposed and designed major steps of MP parameterization of EEG transients and detection of spindles, supervised and contributed to the development of the software, designed the current study and wrote most of the text. PR has written the interactive plugin for detection of structures and display of reports from MP decompositions, fixed the Svarog interface to MP and bugs found during preparation of this study, and consulted mathematical aspects of MP. UM contributed to tests of the software, data analysis and interpretation, drafting of the work and reviewing the manuscript. MZ contributed to tests of the software, tested several detection schemes and performed large part of data analysis and comparisons with visual detections. COR performed MP decompositions on MASS database, performed analyzes, and contributed in writing and reviewing the manuscript. JZ adjusted details of the detection algorithm, supervised the comparison with visual detection, performed cross-validation analyzes, and contributed in writing and reviewing the manuscript.

### Conflict of interest statement

The authors declare that the research was conducted in the absence of any commercial or financial relationships that could be construed as a potential conflict of interest.

## Footnotes

^1^ϵ relates to the maximum distance between two neighboring functions available for decomposition. The distance between two Gabor functions *g*_1_ and *g*_2_ from the dictionary *D*, proposed in Kuś et al. (2013), is measured in the space of inner products 〈*g*_1_|*g*_2_〉 related to the energy as

(4) $$d(g_{1},g_{2})=\sqrt{1-\langle g_{1}|g_{2}\rangle }$$

Dictionary is constructed in such a way that this distance is kept uniform across the neighboring functions. When fitting the dictionary's functions to a signal, the maximum error occurs when a signals structure falls exactly in between two functions available in the dictionary. In such a dictionary, this error will not exceed the distance between neighboring functions from the dictionary. In energy units it will be *d*(*g*_1_, *g*_2_)^2^—the (maximum) “energy error” ϵ.

^2^PPV is related to False Discovery Rate as: PPV = 1 − FDR.

^3^One should be careful on reading of this paper since the authors call false-positive rate what is usually referred to as false detection rate. False positive rate is generally considered as $FPR=\frac{FP}{FP+TN}$ and therefore, as for specificity, due to huge counts of *TN* relative to other counts, is of little interest for characterizing sleep spindle detectors.

^4^Precision is another name for PPV and recall for sensitivity.
